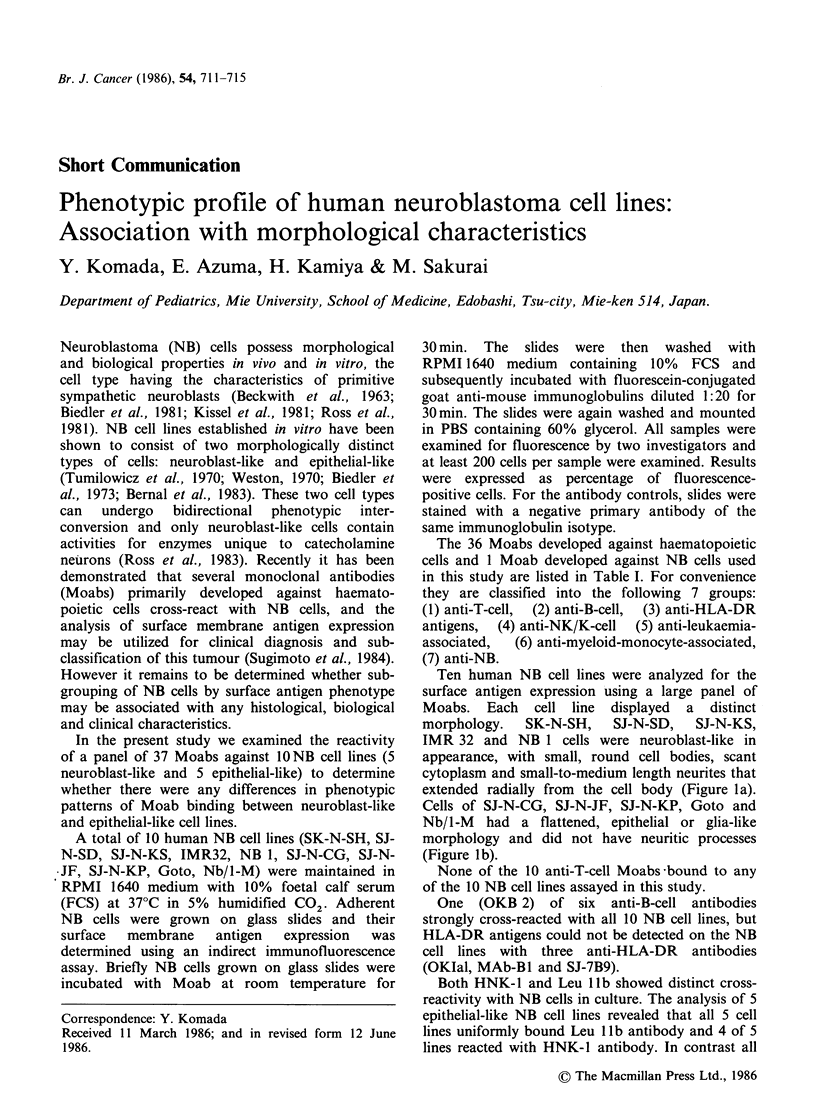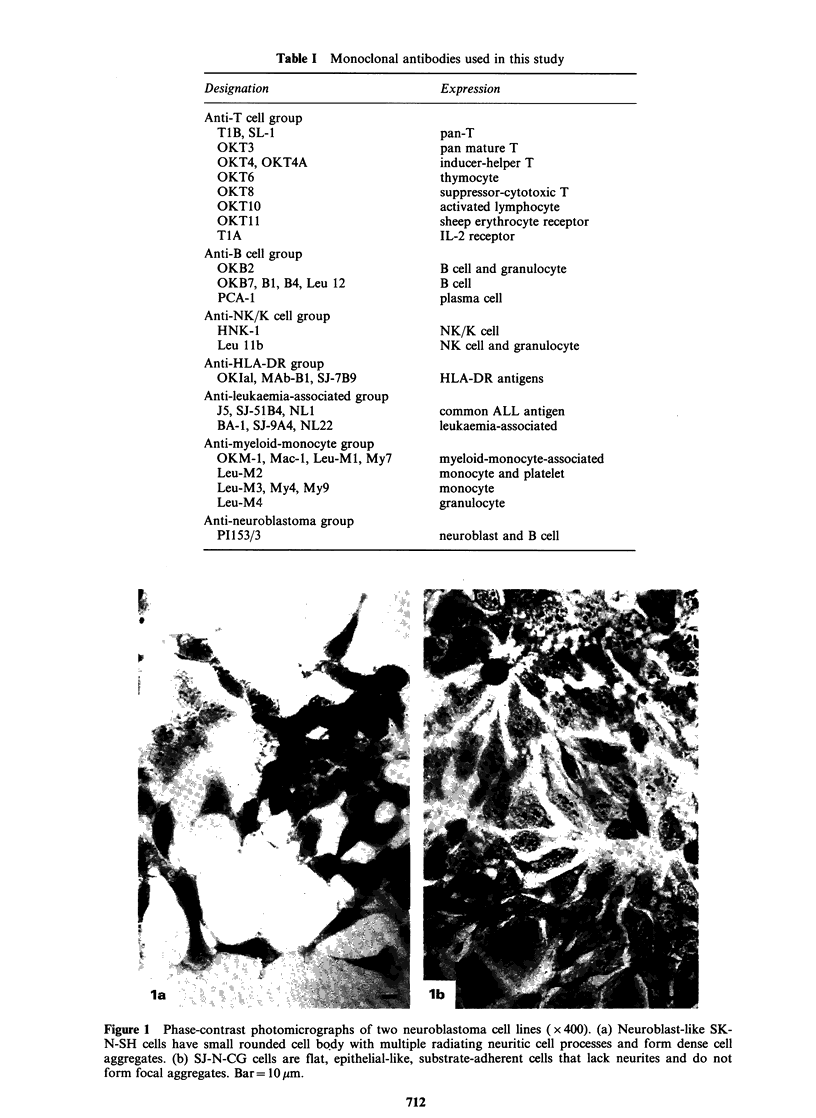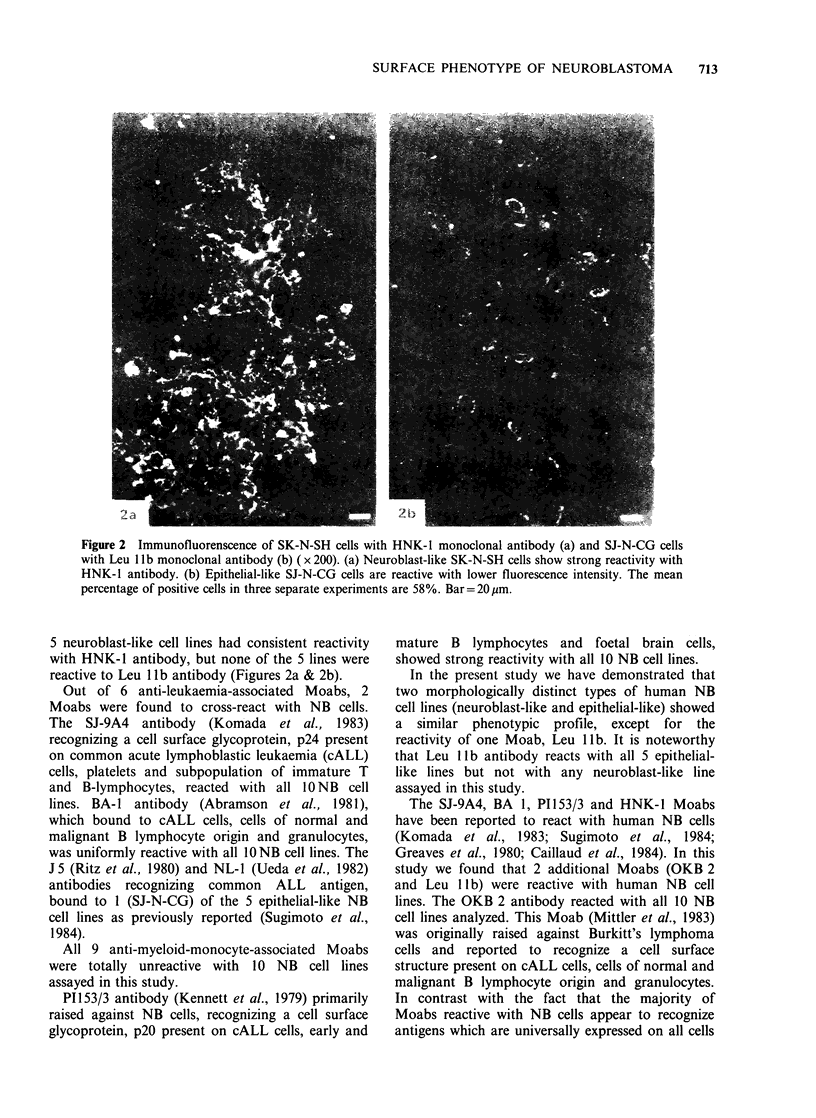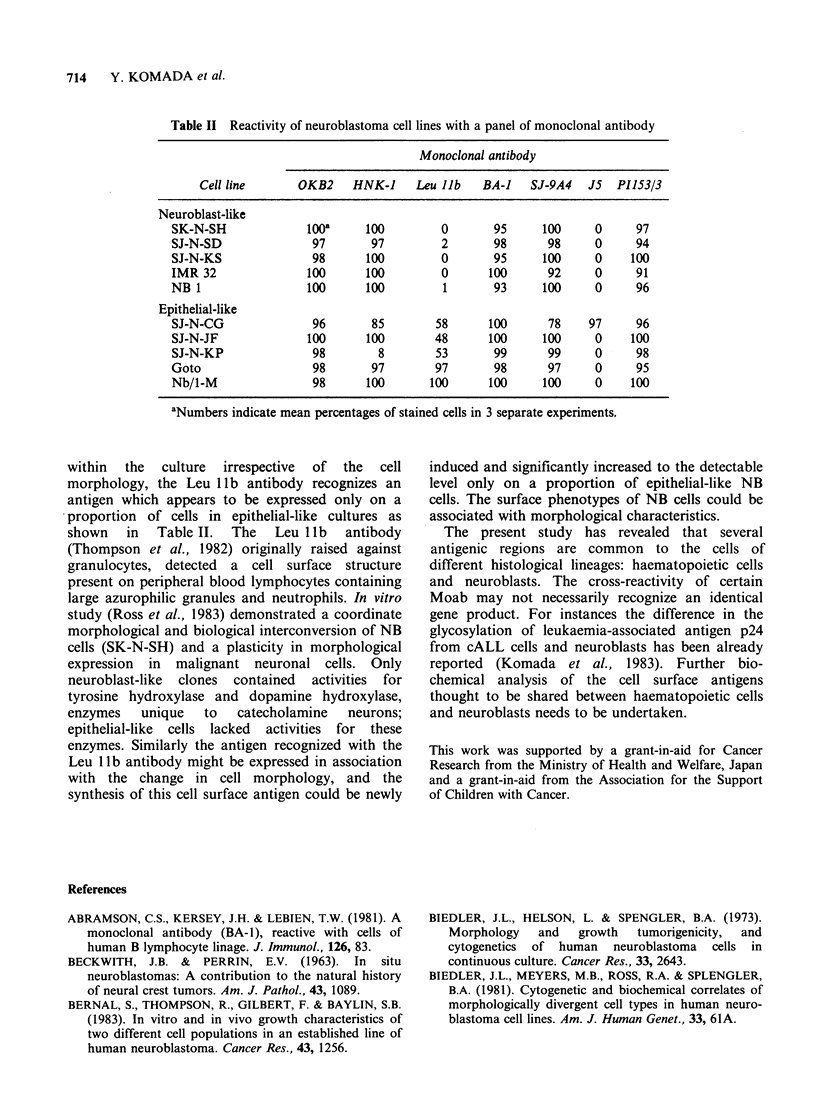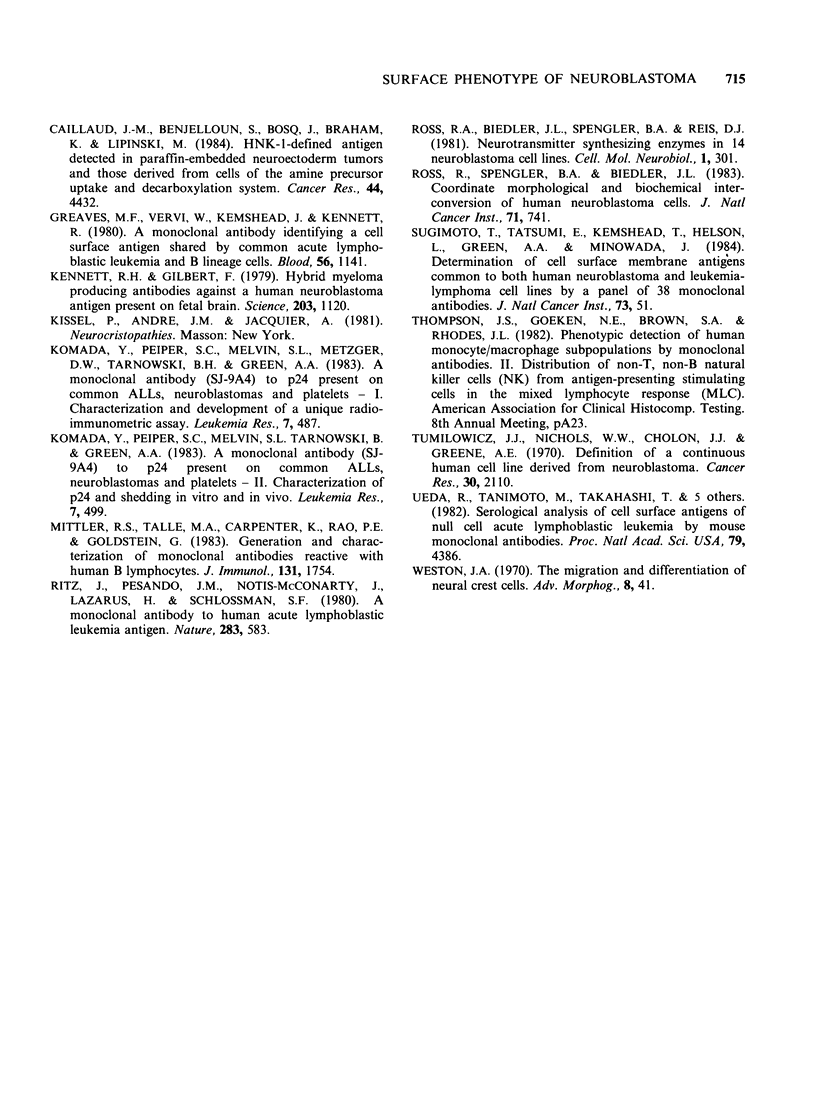# Phenotypic profile of human neuroblastoma cell lines: association with morphological characteristics.

**DOI:** 10.1038/bjc.1986.231

**Published:** 1986-10

**Authors:** Y. Komada, E. Azuma, H. Kamiya, M. Sakurai

## Abstract

**Images:**


Br. J. Cancer (1986), **54**, 711–715

**Short Communication**

### Phenotypic profile of human neuroblastoma cell lines: Association with morphological characteristics

Y. Komada, E. Azuma, H. Kamiya & M. Sakurai

*Department of Pediatrics, Mie University, School of Medicine, Edobashi, Tsu-city, Mie-ken 514, Japan.*

Neuroblastoma (NB) cells possess morphological and biological properties *in vivo* and *in vitro*, the cell type having the characteristics of primitive sympathetic neuroblasts (Beckwith *et al.*, 1963; Biedler *et al.*, 1981; Kissel *et al.*, 1981; Ross *et al.*, 1981). NB cell lines established *in vitro* have been shown to consist of two morphologically distinct types of cells: neuroblast-like and epithelial-like (Tumilowicz *et al.*, 1970; Weston, 1970; Biedler *et al.*, 1973; Bernal *et al.*, 1983). These two cell types can undergo bidirectional phenotypic interconversion and only neuroblast-like cells contain activities for enzymes unique to catecholamine neurons (Ross *et al.*, 1983). Recently it has been demonstrated that several monoclonal antibodies (Moabs) primarily developed against haematopoietic cells cross-react with NB cells, and the analysis of surface membrane antigen expression may be utilized for clinical diagnosis and subclassification of this tumour (Sugimoto *et al.*, 1984). However it remains to be determined whether subgrouping of NB cells by surface antigen phenotype may be associated with any histological, biological and clinical characteristics.

In the present study we examined the reactivity of a panel of 37 Moabs against 10 NB cell lines (5 neuroblast-like and 5 epithelial-like) to determine whether there were any differences in phenotypic patterns of Moab binding between neuroblast-like and epithelial-like cell lines.

A total of 10 human NB cell lines (SK-N-SH, SJ-N-SD, SJ-N-KS, IMR32, NB 1, SJ-N-CG, SJ-N-JF, SJ-N-KP, Goto, Nb/1-M) were maintained in RPMI 1640 medium with 10% foetal calf serum (FCS) at 37°C in 5% humidified $CO_2$. Adherent NB cells were grown on glass slides and their surface membrane antigen expression was determined using an indirect immunofluorescence assay. Briefly NB cells grown on glass slides were incubated with Moab at room temperature for 30 min. The slides were then washed with RPMI 1640 medium containing 10% FCS and subsequently incubated with fluorescein-conjugated goat anti-mouse immunoglobulins diluted 1:20 for 30 min. The slides were again washed and mounted in PBS containing 60% glycerol. All samples were examined for fluorescence by two investigators and at least 200 cells per sample were examined. Results were expressed as percentage of fluorescence-positive cells. For the antibody controls, slides were stained with a negative primary antibody of the same immunoglobulin isotype.

The 36 Moabs developed against haematopoietic cells and 1 Moab developed against NB cells used in this study are listed in Table I. For convenience they are classified into the following 7 groups: (1) anti-T-cell, (2) anti-B-cell, (3) anti-HLA-DR antigens, (4) anti-NK/K-cell (5) anti-leukaemia-associated, (6) anti-myeloid-monocyte-associated, (7) anti-NB.

Ten human NB cell lines were analyzed for the surface antigen expression using a large panel of Moabs. Each cell line displayed a distinct morphology. SK-N-SH, SJ-N-SD, SJ-N-KS, IMR 32 and NB 1 cells were neuroblast-like in appearance, with small, round cell bodies, scant cytoplasm and small-to-medium length neurites that extended radially from the cell body (Figure 1a). Cells of SJ-N-CG, SJ-N-JF, SJ-N-KP, Goto and Nb/1-M had a flattened, epithelial or glia-like morphology and did not have neuritic processes (Figure 1b).

None of the 10 anti-T-cell Moabs bound to any of the 10 NB cell lines assayed in this study.

One (OKB 2) of six anti-B-cell antibodies strongly cross-reacted with all 10 NB cell lines, but HLA-DR antigens could not be detected on the NB cell lines with three anti-HLA-DR antibodies (OKIaI, MAb-B1 and SJ-7B9).

Both HNK-1 and Leu 11b showed distinct cross-reactivity with NB cells in culture. The analysis of 5 epithelial-like NB cell lines revealed that all 5 cell lines uniformly bound Leu 11b antibody and 4 of 5 lines reacted with HNK-1 antibody. In contrast all

Correspondence: Y. Komada
Received 11 March 1986; and in revised form 12 June 1986.

© The Macmillan Press Ltd., 1986

**Table I** Monoclonal antibodies used in this study

| *Designation* | *Expression* |
|---|---|
| Anti-T cell group | |
| T1B, SL-1 | pan-T |
| OKT3 | pan mature T |
| OKT4, OKT4A | inducer-helper T |
| OKT6 | thymocyte |
| OKT8 | suppressor-cytotoxic T |
| OKT10 | activated lymphocyte |
| OKT11 | sheep erythrocyte receptor |
| T1A | IL-2 receptor |
| Anti-B cell group | |
| OKB2 | B cell and granulocyte |
| OKB7, B1, B4, Leu 12 | B cell |
| PCA-1 | plasma cell |
| Anti-NK/K cell group | |
| HNK-1 | NK/K cell |
| Leu 11b | NK cell and granulocyte |
| Anti-HLA-DR group | |
| OKIal, MAb-B1, SJ-7B9 | HLA-DR antigens |
| Anti-leukaemia-associated group | |
| J5, SJ-51B4, NL1 | common ALL antigen |
| BA-1, SJ-9A4, NL22 | leukaemia-associated |
| Anti-myeloid-monocyte group | |
| OKM-1, Mac-1, Leu-M1, My7 | myeloid-monocyte-associated |
| Leu-M2 | monocyte and platelet |
| Leu-M3, My4, My9 | monocyte |
| Leu-M4 | granulocyte |
| Anti-neuroblastoma group | |
| PI153/3 | neuroblast and B cell |

**Figure 1** Phase-contrast photomicrographs of two neuroblastoma cell lines (×400). (a) Neuroblast-like SK-N-SH cells have small rounded cell body with multiple radiating neuritic cell processes and form dense cell aggregates. (b) SJ-N-CG cells are flat, epithelial-like, substrate-adherent cells that lack neurites and do not form focal aggregates. Bar = 10 $\mu$m.

**Figure 2** Immunofluorenscence of SK-N-SH cells with HNK-1 monoclonal antibody (a) and SJ-N-CG cells with Leu 11b monoclonal antibody (b) ( × 200). (a) Neuroblast-like SK-N-SH cells show strong reactivity with HNK-1 antibody. (b) Epithelial-like SJ-N-CG cells are reactive with lower fluorescence intensity. The mean percentage of positive cells in three separate experiments are 58%. Bar = 20 μm.

5 neuroblast-like cell lines had consistent reactivity with HNK-1 antibody, but none of the 5 lines were reactive to Leu 11b antibody (Figures 2a & 2b).

Out of 6 anti-leukaemia-associated Moabs, 2 Moabs were found to cross-react with NB cells. The SJ-9A4 antibody (Komada *et al.*, 1983) recognizing a cell surface glycoprotein, p24 present on common acute lymphoblastic leukaemia (cALL) cells, platelets and subpopulation of immature T and B-lymphocytes, reacted with all 10 NB cell lines. BA-1 antibody (Abramson *et al.*, 1981), which bound to cALL cells, cells of normal and malignant B lymphocyte origin and granulocytes, was uniformly reactive with all 10 NB cell lines. The J 5 (Ritz *et al.*, 1980) and NL-1 (Ueda *et al.*, 1982) antibodies recognizing common ALL antigen, bound to 1 (SJ-N-CG) of the 5 epithelial-like NB cell lines as previously reported (Sugimoto *et al.*, 1984).

All 9 anti-myeloid-monocyte-associated Moabs were totally unreactive with 10 NB cell lines assayed in this study.

PI153/3 antibody (Kennett *et al.*, 1979) primarily raised against NB cells, recognizing a cell surface glycoprotein, p20 present on cALL cells, early and mature B lymphocytes and foetal brain cells, showed strong reactivity with all 10 NB cell lines.

In the present study we have demonstrated that two morphologically distinct types of human NB cell lines (neuroblast-like and epithelial-like) showed a similar phenotypic profile, except for the reactivity of one Moab, Leu 11b. It is noteworthy that Leu 11b antibody reacts with all 5 epithelial-like lines but not with any neuroblast-like line assayed in this study.

The SJ-9A4, BA 1, PI153/3 and HNK-1 Moabs have been reported to react with human NB cells (Komada *et al.*, 1983; Sugimoto *et al.*, 1984; Greaves *et al.*, 1980; Caillaud *et al.*, 1984). In this study we found that 2 additional Moabs (OKB 2 and Leu 11b) were reactive with human NB cell lines. The OKB 2 antibody reacted with all 10 NB cell lines analyzed. This Moab (Mittler *et al.*, 1983) was originally raised against Burkitt's lymphoma cells and reported to recognize a cell surface structure present on cALL cells, cells of normal and malignant B lymphocyte origin and granulocytes. In contrast with the fact that the majority of Moabs reactive with NB cells appear to recognize antigens which are universally expressed on all cells

**Table II** Reactivity of neuroblastoma cell lines with a panel of monoclonal antibody

| Cell line | *Monoclonal antibody* | | | | | | |
|---|---|---|---|---|---|---|---|
| | *OKB2* | *HNK-1* | *Leu 11b* | *BA-1* | *SJ-9A4* | *J5* | *PI153/3* |
| Neuroblast-like | | | | | | | |
| SK-N-SH | 100[a] | 100 | 0 | 95 | 100 | 0 | 97 |
| SJ-N-SD | 97 | 97 | 2 | 98 | 98 | 0 | 94 |
| SJ-N-KS | 98 | 100 | 0 | 95 | 100 | 0 | 100 |
| IMR 32 | 100 | 100 | 0 | 100 | 92 | 0 | 91 |
| NB 1 | 100 | 100 | 1 | 93 | 100 | 0 | 96 |
| Epithelial-like | | | | | | | |
| SJ-N-CG | 96 | 85 | 58 | 100 | 78 | 97 | 96 |
| SJ-N-JF | 100 | 100 | 48 | 100 | 100 | 0 | 100 |
| SJ-N-KP | 98 | 8 | 53 | 99 | 99 | 0 | 98 |
| Goto | 98 | 97 | 97 | 98 | 97 | 0 | 95 |
| Nb/1-M | 98 | 100 | 100 | 100 | 100 | 0 | 100 |

[a]Numbers indicate mean percentages of stained cells in 3 separate experiments.

within the culture irrespective of the cell morphology, the Leu 11b antibody recognizes an antigen which appears to be expressed only on a proportion of cells in epithelial-like cultures as shown in Table II. The Leu 11b antibody (Thompson *et al.*, 1982) originally raised against granulocytes, detected a cell surface structure present on peripheral blood lymphocytes containing large azurophilic granules and neutrophils. *In vitro* study (Ross *et al.*, 1983) demonstrated a coordinate morphological and biological interconversion of NB cells (SK-N-SH) and a plasticity in morphological expression in malignant neuronal cells. Only neuroblast-like clones contained activities for tyrosine hydroxylase and dopamine hydroxylase, enzymes unique to catecholamine neurons; epithelial-like cells lacked activities for these enzymes. Similarly the antigen recognized with the Leu 11b antibody might be expressed in association with the change in cell morphology, and the synthesis of this cell surface antigen could be newly induced and significantly increased to the detectable level only on a proportion of epithelial-like NB cells. The surface phenotypes of NB cells could be associated with morphological characteristics.

The present study has revealed that several antigenic regions are common to the cells of different histological lineages: haematopoietic cells and neuroblasts. The cross-reactivity of certain Moab may not necessarily recognize an identical gene product. For instances the difference in the glycosylation of leukaemia-associated antigen p24 from cALL cells and neuroblasts has been already reported (Komada *et al.*, 1983). Further biochemical analysis of the cell surface antigens thought to be shared between haematopoietic cells and neuroblasts needs to be undertaken.

This work was supported by a grant-in-aid for Cancer Research from the Ministry of Health and Welfare, Japan and a grant-in-aid from the Association for the Support of Children with Cancer.

### References

ABRAMSON, C.S., KERSEY, J.H. & LEBIEN, T.W. (1981). A monoclonal antibody (BA-1), reactive with cells of human B lymphocyte linage. *J. Immunol.*, **126**, 83.

BECKWITH, J.B. & PERRIN, E.V. (1963). In situ neuroblastomas: A contribution to the natural history of neural crest tumors. *Am. J. Pathol.*, **43**, 1089.

BERNAL, S., THOMPSON, R., GILBERT, F. & BAYLIN, S.B. (1983). In vitro and in vivo growth characteristics of two different cell populations in an established line of human neuroblastoma. *Cancer Res.*, **43**, 1256.

BIEDLER, J.L., HELSON, L. & SPENGLER, B.A. (1973). Morphology and growth tumorigenicity, and cytogenetics of human neuroblastoma cells in continuous culture. *Cancer Res.*, **33**, 2643.

BIEDLER, J.L., MEYERS, M.B., ROSS, R.A. & SPLENGLER, B.A. (1981). Cytogenetic and biochemical correlates of morphologically divergent cell types in human neuroblastoma cell lines. *Am. J. Human Genet.*, **33**, 61A.

CAILLAUD, J.-M., BENJELLOUN, S., BOSQ, J., BRAHAM, K. & LIPINSKI, M. (1984). HNK-1-defined antigen detected in paraffin-embedded neuroectoderm tumors and those derived from cells of the amine precursor uptake and decarboxylation system. *Cancer Res.*, **44,** 4432.

GREAVES, M.F., VERVI, W., KEMSHEAD, J. & KENNETT, R. (1980). A monoclonal antibody identifying a cell surface antigen shared by common acute lymphoblastic leukemia and B lineage cells. *Blood,* **56,** 1141.

KENNETT, R.H. & GILBERT, F. (1979). Hybrid myeloma producing antibodies against a human neuroblastoma antigen present on fetal brain. *Science,* **203,** 1120.

KISSEL, P., ANDRE, J.M. & JACQUIER, A. (1981). *Neurocristopathies.* Masson: New York.

KOMADA, Y., PEIPER, S.C., MELVIN, S.L., METZGER, D.W., TARNOWSKI, B.H. & GREEN, A.A. (1983). A monoclonal antibody (SJ-9A4) to p24 present on common ALLs, neuroblastomas and platelets – I. Characterization and development of a unique radioimmunometric assay. *Leukemia Res.*, **7,** 487.

KOMADA, Y., PEIPER, S.C., MELVIN, S.L. TARNOWSKI, B. & GREEN, A.A. (1983). A monoclonal antibody (SJ-9A4) to p24 present on common ALLs, neuroblastomas and platelets – II. Characterization of p24 and shedding in vitro and in vivo. *Leukemia Res.*, **7,** 499.

MITTLER, R.S., TALLE, M.A., CARPENTER, K., RAO, P.E. & GOLDSTEIN, G. (1983). Generation and characterization of monoclonal antibodies reactive with human B lymphocytes. *J. Immunol.*, **131,** 1754.

RITZ, J., PESANDO, J.M., NOTIS-McCONARTY, J., LAZARUS, H. & SCHLOSSMAN, S.F. (1980). A monoclonal antibody to human acute lymphoblastic leukemia antigen. *Nature,* **283,** 583.

ROSS, R.A., BIEDLER, J.L., SPENGLER, B.A. & REIS, D.J. (1981). Neurotransmitter synthesizing enzymes in 14 neuroblastoma cell lines. *Cell. Mol. Neurobiol.*, **1,** 301.

ROSS, R., SPENGLER, B.A. & BIEDLER, J.L. (1983). Coordinate morphological and biochemical interconversion of human neuroblastoma cells. *J. Natl Cancer Inst.*, **71,** 741.

SUGIMOTO, T., TATSUMI, E., KEMSHEAD, T., HELSON, L., GREEN, A.A. & MINOWADA, J. (1984). Determination of cell surface membrane antigens common to both human neuroblastoma and leukemia-lymphoma cell lines by a panel of 38 monoclonal antibodies. *J. Natl Cancer Inst.*, **73,** 51.

THOMPSON, J.S., GOEKEN, N.E., BROWN, S.A. & RHODES, J.L. (1982). Phenotypic detection of human monocyte/macrophage subpopulations by monoclonal antibodies. II. Distribution of non-T, non-B natural killer cells (NK) from antigen-presenting stimulating cells in the mixed lymphocyte response (MLC). American Association for Clinical Histocomp. Testing. 8th Annual Meeting, pA23.

TUMILOWICZ, J.J., NICHOLS, W.W., CHOLON, J.J. & GREENE, A.E. (1970). Definition of a continuous human cell line derived from neuroblastoma. *Cancer Res.*, **30,** 2110.

UEDA, R., TANIMOTO, M., TAKAHASHI, T. & 5 others. (1982). Serological analysis of cell surface antigens of null cell acute lymphoblastic leukemia by mouse monoclonal antibodies. *Proc. Natl Acad. Sci. USA,* **79,** 4386.

WESTON, J.A. (1970). The migration and differentiation of neural crest cells. *Adv. Morphog.*, **8,** 41.